# Generation of single stranded DNA with selective affinity to bovine spermatozoa

**DOI:** 10.5713/ajas.20.0235

**Published:** 2020-08-21

**Authors:** Sivadasan Pathiyil Vinod, Rajamani Vignesh, Mani Priyanka, Krishnaswamy Gopalan Tirumurugaan, Salem Nagalingam Sivaselvam, Gopal Dhinakar Raj

**Affiliations:** 1 Department of Animal Biotechnology, Madras Veterinary College, Tamil Nadu Veterinary and Animal Sciences University, Chennai – 600051, India; 2 Department of Animal Genetics and Breeding, Madras Veterinary College, Tamil Nadu Veterinary and Animal Sciences University, Chennai – 600051, India; 3 Centre for Animal Health Studies, Tamil Nadu Veterinary and Animal Sciences University, Chennai – 600051, India

**Keywords:** Aptamers, Binding, Selective Enrichment, Surface Characteristics

## Abstract

**Objective:**

This study was conducted to generate single stranded DNA oligonucleotides with selective affinity to bovine spermatozoa, assess its binding potential and explore its potential utility in trapping spermatozoa from suspensions.

**Methods:**

A combinatorial library of 94 mer long oligonucleotide was used for systematic evolution of ligands by exponential enrichment (SELEX) with bovine spermatozoa. The amplicons from sixth and seventh rounds of SELEX were sequenced, and the reads were clustered employing cluster database at high identity with tolerance (CD-HIT) and FASTAptamer. The enriched nucleotides were predicted for secondary structures by Mfold, motifs by Multiple Em for Motif Elicitation and 5′ labelled with biotin/6-FAM to determine the binding potential and binding pattern.

**Results:**

We generated 14.1 and 17.7 million reads from sixth and seventh rounds of SELEX respectively to bovine spermatozoa. The CD-HIT clustered 78,098 and 21,196 reads in the top ten clusters and FASTAptamer identified 2,195 and 4,405 unique sequences in the top three clusters from the sixth and seventh rounds, respectively. The identified oligonucleotides formed secondary structures with delta G values between −1.17 to −26.18 kcal/mol indicating varied stability. Confocal imaging with the oligonucleotides from the seventh round revealed different patterns of binding to bovine spermatozoa (fluorescence of the whole head, spot of fluorescence in head and mid- piece and tail). Use of a 5′-biotin tagged oligonucleotide from the sixth round at 100 pmol with 4×10^6^ spermatozoa could trap almost 80% from the suspension.

**Conclusion:**

The binding patterns and ability of the identified oligonucleotides confirms successful optimization of the SELEX process and generation of aptamers to bovine spermatozoa. These oligonucleotides provide a quick approach for selective capture of spermatozoa from complex samples. Future SELEX rounds with X- or Y- enriched sperm suspension will be used to generate oligonucleotides that bind to spermatozoa of a specific sex type.

## INTRODUCTION

The growing demand of food for the human population that is predicted to reach ten billion by 2050 necessitates the use of modern biotechnology tools for sustainable production from both animal and agricultural resources. Manipulating the sex ratio would provide important economic benefits for profitable livestock farming and would accelerate the improvement of genetic potential. The use of sperm or embryo sexing along with other ‘Omics’ approaches is a recognized and much sought-after approach to produce pre-sexed livestock. There have been several attempts to exploit the unique features of the X- and Y- spermatozoa such as male specific H-Y antigen [1,2], antibodies to female sex-specific proteins [2], surface charges [3], difference in spermatozoa head volume [4] and differences in the DNA content [5] and utilize them to increase the comparative percentage of X- or Y-spermatozoa.

Developing molecules with specific affinity to spermatozoa would help to enhance understanding of the sperm biology as well as help to generate information on the subtle surface differences of the spermatozoa. Short ribonucleic acid (RNA) and single-strand deoxyribonucleic acid (ssDNA) or peptides are an interesting and alternate class of molecules which can bind to their targets with high affinity and specificity due to their specific three-dimensional structures [6,7]. These single stranded molecules fold under native conditions and generate structures that are capable of binding specifically to ligands leading to the generation of a new class of molecules termed as aptamers (‘*aptus*’ meaning ‘to fit’ and ‘*meros*’ meaning ‘part’ in Latin). Thus aptamers are alternative equivalents to antibodies that are generated by the process of systematic evolution of ligands by exponential enrichment (SELEX) employing high affinity pools of variant sequences of nucleic acid ligands for proteins or other immunological/non-immunological structures and amplification of the bound species [6,7].

These short oligonucleotides are shown to have great potential due to their small size, high specificities, and their potential ability to differentiate between splice variants and post transcriptional modification of the same protein [8]. In addition, a binding affinity of 10^4^ times without cross reactivity [9], dissociation constants from micromoles to nanomoles and even picomoles [10], ease of production, scale up, and modification *in-vitro*, amenability to reverse genetic techniques, high stability and ability to renature after denaturation and storage at ambient conditions are other potential advantages. In addition, oligonucleotides can also be generated against non-protein structures thereby providing opportunity to understand the contribution of non-protein structures to membrane biology. Aptamers have been generated for various biomedical application including therapeutics [11–14], aptasensors [15], biosensors [16, 17], diagnostic [18,19], and imaging systems [20]. Efforts have been made to generate X- and Y- sex-specific oligonucleotide from a large randomised library to generate oligonucleotides that selectively bind to boar X- or Y- spermatozoa [21]. Due to above inherent advantages, as a first step we choose to use a random oligonucleotide library, perform SELEX rounds with bovine spermatozoa and identify oligonucleotides that exhibit binding to bovine spermatozoa. A successful attempt in this approach might provide an optimized protocol that could be applied for generation of alternate molecules with other specificities to bovine spermatozoa with potential downstream applications.

## MATERIALS AND METHODS

### Oligo library, sense, and anti-sense primers for generation of oligonucleotides with affinity

A ssDNA oligonucleotide library with a central random 40 nucleotide (nt) region (that has almost 2.56 million types of oligonucleotide combinations) flanked on either side by 27 nucleotides to serve as primer binding sites (Oligonucleotide library: *5*′*-TCCATCTCTTCTGTATGTCGAGATCTA-40N [A/G/C/T)-TAGATCTCCTAACCGACTCCGTTATTT-3*′; primer pairs AptaF: *5*′*-TCCATCTC TTCTGTATGTCGAG ATCTA-3*′ and Apta R: 5′-GATTAACGGAGTCGGTTAG GAGATCTA-3′) was designed. The 27-nucleotide primer binding site was BLAST verified against *Bos* (taxid: 9903) to confirm that the entire query length did not provide any similarity and to ensure that the oligonucleotides bound to the spermatozoa membrane are only amplified during the SELEX rounds and not the free nucleic acid from bovine spermatozoa]. The forward (sense) primer and reverse (anti-sense) primers complementary to the known 27 nucleotides were synthesised with/without 5′ modification for use in specific steps in the study (unlabelled primers for the SELEX rounds and FAM/biotin labelled primers for the localization and binding studies).

### Polymerase chain reaction amplification of the oligonucleotides library and generation of single stranded DNA

The basic requirement for SELEX was to generate ssDNA that forms different secondary structures at ambient temperature and enable specific binding with the protein/structure of interest. The synthesized oligonucleotide library was amplified using Vent polymerase (New England Biolabs, Ipswich, MA, USA) and the optimization included the annealing temperature, the ratio of the forward: reverse primer (1:1 to 60:1), number of polymerase chain reaction (PCR) cycles (15,20, 25, and 30 cycles) and template concentrations (0.5 to 7 pmol) to generate the single strand of interest by asymmetric PCR. The PCR amplification cycle included 94°C for 5 minutes, 29 cycles each of 94°C for 30 seconds, temperature range 50°C to 67°C for 30 seconds and 72°C for 30 seconds with a final extension at 72°C for 5 minutes. Following PCR, the amplicons were electrophoresed in a 4% agarose gel in Tris borate ethylenediaminetetraacetic acid (TBE) buffer with Safe View Classic dye (Applied Biological Materials Inc., Richmond, Canada) at 50 V/cm and the results were documented.

### SELEX rounds to enrich oligonucleotides with binding potential to spermatozoa

Frozen bovine (Jersey cross) semen straws of five different bulls were thawed, characterized for their count (using Haemocytometer), motility (microscopic) and acrosome integrity (microscopic) following standard procedures. For every round of SELEX, the semen straws from these bulls were thawed pooled, spermatozoa were pelleted by centrifugation at 450 g for 5 minutes and washed twice with wash and binding buffer (WBB-Sterile phosphate buffer saline supplemented with glucose to a final concentration of 5 mM). A 500 μL aliquot of WBB with 2×10^8^ spermatozoa was used for binding with the oligonucleotide library (at the specified concentration) at for one hour at room temperature. The unbound oligonucleotides were removed and the spermatozoa with the bound oligonucleotides were lysed at 56°C for 1 hour with 20 μL of lysis buffer 1 (containing 20 mM dithiothreitol, 1.7 μM sodium dodecyl sulphate, 1 mg/mL proteinase K in 1× ThermoPol Buffer [NEB, Ipswich, MA, USA]), inactivated at 95°C for 10 minutes and mixed with 10 μL of lysis buffer 2 (50 mM dithiothreitol, 200 mM potassium hydroxide). This mixture was again incubated at 65°C for 10 minutes and finally neutralized with 10 μL of neutralising buffer (300 mM potassium chloride and 900 mM of Tris HCl, pH 8.3). The lysate thus prepared was used as template in PCR to generate the ssDNA by asymmetric PCR which used in the subsequent rounds of SELEX. A total of six rounds of positive SELEX with bovine spermatozoa and a round of negative SELEX with the diluent and the tubes used for binding were performed and the purified amplicons from the sixth and seventh rounds of SELEX were sequenced in Illumina Hiseq 2500 platform (at Clevergene Biocorp Pvt. Ltd., Bangalore, India).

### Bioinformatic analysis

**Figure 1:**
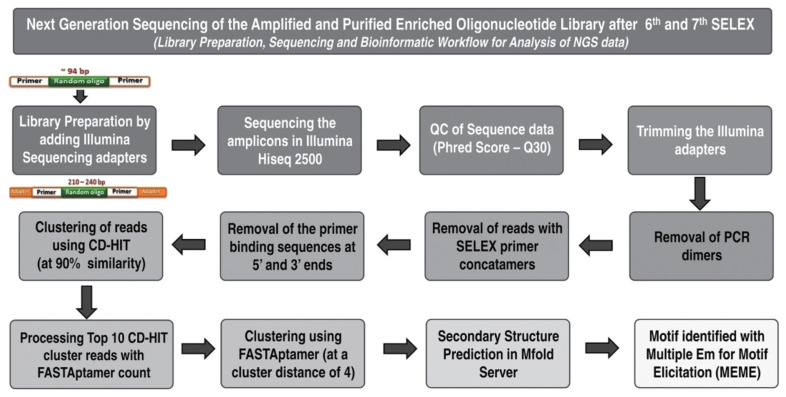
Overall approach for the next generation sequencing (NGS) of the amplified and purified enriched oligonucleotide library after 6th and 7th rounds of SELEX: The approached followed for library preparation, sequencing and bioinformatic work flow to analyze the generated NGS data is provided. SELEX, systematic evolution of ligands by exponential enrichment.

The overall bioinformatic workflow is depicted in the Figure 1. Following sequencing, the quality passed reads (Phred score) were trimmed, clustered at 90% sequence similarity using cluster database at high identity with tolerance-EST (CD-HIT-EST) and the sequences in the top 10 CD-HIT cluster sequences were counted and clustered again using FASTAptamer count module in the FASTAptamer software (with the Levenshtein edit distance parameter set to 4). Following the identification of enriched sequences using FASTAptamer, the Multiple Em for Motif Elicitation (MEME at http://meme-suite.org/tools/meme) tool was used to identify motifs. The secondary structures formed by these enriched sequences were identified in the mfold web server (http://unafold.rna.albany.edu/?q=mfold/DNA-Folding-Form). The representative sequences in the top three clusters from the sixth and seventh rounds of SELEX were synthesized with 5′ modifications (Biotin/FAM tag respectively) to determine their binding potential to bovine spermatozoa.

### Characterization of enriched oligonucleotides for their binding to spermatozoa

The binding of the selected oligonucleotides to bovine spermatozoa was determined in two different experiments.

#### Experiment 1

The 5′ biotin modified oligonucleotides (identified from sixth round- R6-Oligo-1; R6-Oligo-2; R6-Oligo-3) were incubated at different concentrations (50,100, and 200 pmol) with 4×10^6^ processed spermatozoa (thawed frozen semen samples, centrifuged and washed with WBB) in a total volume of 500 μL for 30 mins. The spermatozoa suspension was washed to remove the unbound oligonucleotides and incubated with a fixed concentration of streptavidin coupled magnetic beads for 30 mins. After incubation, the spermatozoa bound with the oligonucleotides were separated under magnetic field and their counts determined using a haemocytometer.

#### Experiment 2

The 5′ 6-FAM modified oligonucleotides (identified from seventh round- cluster_1869, cluster_5 and cluster_2015) were incubated at 20 pmol concentration (individually or all three combined together) with 4×10^6^ processed spermatozoa in a total volume of 500 μL. After removal of the unbound nucleotides by centrifugation, the spermatozoa were counter stained with 4′, 6-diamidino-2-phenylindole. The spermatozoa suspension was washed, an aliquot was placed on a glass slide, air dried, mounted with ProLong Glass Antifade Mountant (ThermoFisher Scientific, Chennai, India) and imaged in a confocal microscope to study the binding pattern of the oligonucleotides.

## RESULTS

### Generation of ssDNA oligonucleotide library by asymmetric polymerase chain reaction

**Figure 2:**
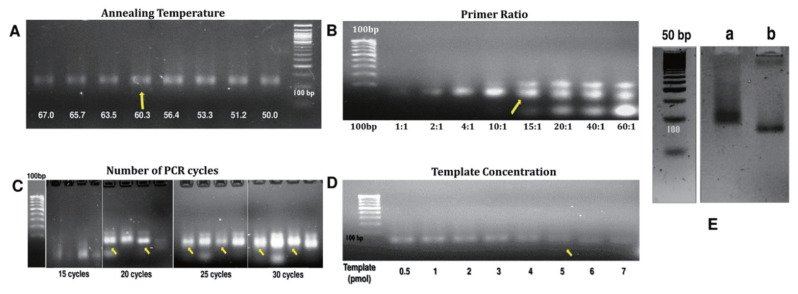
Optimization of the PCR conditions for amplification of the 94 mer randomized oligonucleotide library as template: The PCR conditions for amplification of the 94 mer oligonucleotide library was optimized before performing the SELEX rounds. (A) Optimization of the annealing temperature: Electrophoresis of the PCR amplicons generated at different annealing temperatures 50°C to 68°C; (B) Optimization of the primer ratio: Electrophoresis of the PCR amplicons generated with biotin modified forward and reverse primer in the ratio as indicated in the image on a 4% agarose gel; (C) Optimization of the number of PCR cycles: Electrophoresis of the PCR amplicons generated with different number of PCR cycles 15, 20, 25, and 30 respectively, on 4% agarose gel; (D) Optimization of the template concentration: The PCR amplicons generated with varied template concentration as indicated in the image on a 4% agarose gel; (E) Representative gel depicting the optimized asymmetric PCR for generation of the ssDNA amplicons: M, 50 bp DNA ladder; Lane a, regular PCR (dsDNA amplicons); Lane b, ssDNA amplicons generated by asymmetric PCR with the optimized conditions listed above. Note: The yellow arrow indicates the optimized conditions for the PCR amplification of the oligonucleotide library. PCR, polymerase chain reaction.

The ssDNA amplicons are slightly smaller when compared to dsDNA amplicons. The primary optimization of the annealing temperature indicated significant amplification at 60.3°C (Figure 2A) and this temperature was selected for optimization of the asymmetric PCR with AptaF/AptaR primers in the ratio between 1:10 to 60:1. As the ratio of primers increased from 15:1 to 60:1, we could observe amplicons at ~100 bp, less than 100 bp and unused primers (Figure 2B). The concentration of the ssDNA increased with the increase in AptaF concentration and the ratio of 10:1 which resulted in satisfactory amplification of ssDNA was selected to optimize the number of PCR cycles and the template concentration. With the use of primers at a ratio of 10:1 significant ssDNA amplification was observed with 30 cycles of PCR and at a template concentration of 7 pmol (Figure 2C, 2D). The optimized template concentration, primer ratio and number of PCR cycles that resulted in optimum ssDNA amplification were used in the different SELEX rounds (Figure 2E).

### Concentration of ssDNA generated in different rounds of SELEX

The first round of SELEX was initiated with using 200 pmol of the oligonucleotide library and it resulted in 416 ng (~6.5 pmol in a total volume of 60 μL) of ssDNA oligonucleotides (Table 1). The yield of the ssDNA library was 9.45, 37.5, 10.31, 12.3, 25.54, and 91.4 pmol in the SELEX rounds from two to seven respectively (in a total volume of 60 μL). In terms of percent increase, a ssDNA concentration of 257.8% was achieved at the seventh SELEX round with an overall enrichment of 13.06 fold across the SELEX rounds.

### Bioinformatic analysis and identification of the enriched ssDNA oligonucleotides

**Figure 3:**
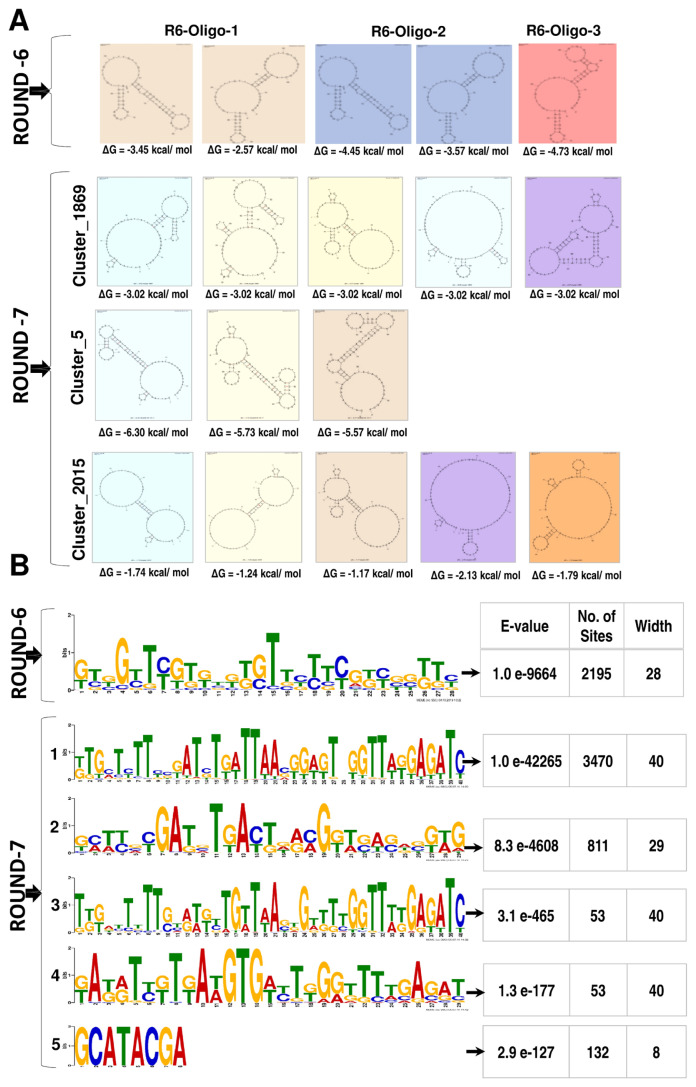
Secondary structure prediction and motif identification by multiple Em for Motif Elicitation (MEME) for the oligonucleotides clustered by FASTAptamer analysis from the sixth and seventh SELEX rounds. The secondary structure was predicted by mfold web server available at http://unafold.rna.albany.edu/?q=mfold/DNA-Folding-Form. (A) Predicted Secondary structures: For the oligonucleotides identified in the Round 6 we could obtain 2, 2, and 1 secondary structures respectively for the R6-Oligo-1, R6-Oligo-2, and R6-Oligo-3 and their ΔG ranged from −2.57 to 4.73 kcal/mol; for the oligonucleotides identified in the round 7 we could obtain 5, 3, and 5 secondary structures respectively for the cluster_1869, cluster_5 and cluster_2015 and their ΔG ranged from −1.17 to −6.30 kcal/mol. (B) The motifs predicted by MEME (available at http://meme-suite.org) analysis for the oligonucleotides identified by the FASTAptamer from the sixth (one motif) and seventh rounds (five different motifs) of SELEX. The insert table provides the details on the motifs present in the number of oligonucleotide sequences/sites from the sixth and seventh round of SELEX. The lower E-value indicates the statistical significance of the identified motif as well as an estimate of how each occurrence (site p-value) matches the motif.

We generated 14.11 and 17.77 million quality passed sequence reads in the Illumina platform with a base calling accuracy of 94 percent at Q30 (Phred score) by sequencing the amplicons of the sixth and seventh rounds of SELEX, respectively. These reads were adapter trimmed and clustered by CD-HIT resulting in 7.38 and 16.9 million clusters and the top 10 CD-HIT clusters contained 78,098 and 21,196 reads from the sixth and seventh SELEX rounds respectively. Among the sequences in the top 10 clusters, 15,701 (20.12%) and 3,113 (14.68%) unique reads were present respectively in the top three clusters (Table 2). FASTAptamer clustering of the reads in the top 10 clusters resulted in 2,195 and 4,405 reads respectively from the sixth and seventh SELEX rounds. The secondary structures predicted using the Mfold server for the representative sequences in the top three clusters revealed ΔG values ranging between −2.57 to −26.18 kcal/mol (Figure 3A). Following the removal of the primer sequences, the MEME algorithm predicted one motif (in 2,195 sequences) and five motifs (in 4,519 sequences of which the first motif was found in 3,470 reads with an E-value of 1.5e-42265) in the sixth and seventh rounds of SELEX respectively (Figure 3B).

### Characterization of the enriched oligonucleotides for their binding to spermatozoa

#### Experiment 1

**Figure 4:**
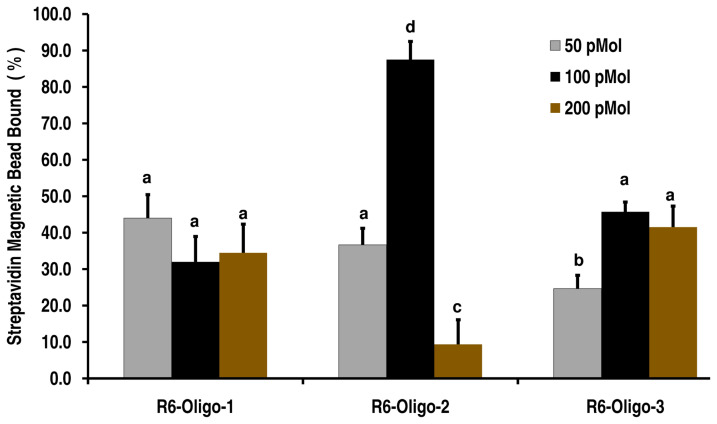
Characterization of enriched oligonucleotides (from round 6) for its binding potential to bovine spermatozoa. The oligonucleotides identified (R6-Oligo-1, R6-Oligo-2, and R6-Oligo-3) were assessed for its binding and ability to trap spermatozoa from a suspension by using streptavidin coupled magnetic beads. The oligonucleotides were 5′ biotin tagged and used 50, 100, and 200 pmol concentration with bovine spermatozoa. The R6-Oligo-2 at 100 pmol concentration resulted in trapping of almost 80% of the spermatozoa indicating a higher binding capability. ^a–d^ Means are different at p<0.05.

Among the three oligonucleotides, R6-Oligo-1 did not reveal any significant difference in the number of spermatozoa captured over the three different concentrations tested (30% to 40%). The R6-Oligo-3 resulted in similar binding efficiency to that of R6-Oligo-1 at 100 and 200 pmol concentrations while at 50 pmol the percentage of spermatozoa bound was only 25%. The R6-Oligo-2 exhibited different binding efficiencies of 35% at 50 pmol, ~20% at 200 pmol and around 80% at 100 pmol concentration (Figure 4).

#### Experiment 2

**Figure 5:**
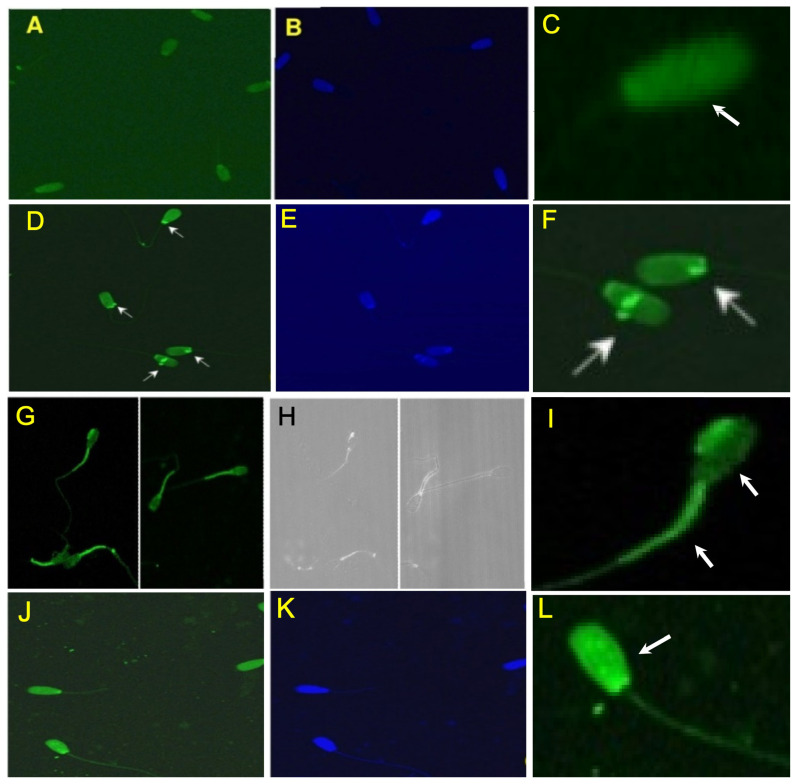
Characterization of enriched oligonucleotides (from round 7) for its binding pattern to bovine spermatozoa determined by confocal imaging. The potential identified enriched oligonucleotides (cluster_1869, cluster_5, and cluster_2015) were labelled at their 5′ end with 6-FAM which enabled visualization of the binding pattern (imaged with 20X Plan-Apochromat" 20x/1.0 DICM27; 70 mm objective). (A), (B) Binding pattern of cluster_1869; (D), (E) Binding pattern of cluster_5; (G), (H) Binding pattern of cluster_2015; (J), (K) Binding pattern with the combination of all the three oligonucleotides; (C), (F), (I), (L) insert of a single spermatozoa representing the pattern of the oligonucleotide binding with the oligonucleotide used. Note: Binding is visible as a bright white spot in B/w image.

The binding patterns of the three different oligonucleotides (cluster_1869, cluster_5, and cluster_2015; 5′ 6-FAM tagged) from the SLEX round 7 were localized by confocal microscopy. The cluster_1869 resulted in complete fluorescence of spermatozoa head. However, the fluorescence was not uniformly distributed in fields observed (Figure 5A–5C). The cluster_5 resulted in mild fluorescence of the head with bright pinpoint green fluorescence. This type of binding was also observed only in a few of the fields screened (Figure 5D–5F). The cluster_2015 resulted in bright pinpoint green fluorescence in the head as well bright green fluorescence in the mid piece and portion of the tail. This type of binding was also observed in many of the fields screened (Figure 5G–5I). Binding of the bovine spermatozoa with a mixture of the oligonucleotides (cluster_1869, cluster_5, and cluster_2015 labelled with 6-FAM) resulted in complete fluorescence of the head, mid piece and portion of the tail as expected. The fluorescence was not uniformly distributed in the fields screened (Figure 5J–5L).

## DISCUSSION

Molecules with greater binding affinity to targets (proteins, receptors and biomarker molecules on cell surface) generated through the process of SELEX, from huge combinatorial oligonucleotide libraries are called aptamers and they have emerged as potential tool for application in different fields. Among the approach/targets to generate aptamers, the Cell-SELEX approach enables identification of aptamers to live cells as targets. The report on expression levels of sperm membrane proteins relating to fertility has opened the window to generate antibodies or similar molecules (single stranded oligonucleotides) with potential binding to these targets. However, data is scarce on the application of SELEX process to generate molecules with affinity to the physical and structural features of bovine spermatozoa. Hence, in this study we bound a random oligonucleotide library to bovine spermatozoa and identified the enriched oligonucleotides with differential binding abilities as assessed by its localization and their ability to trap bovine spermatozoa from a suspension.

The width of spermatozoa head across different livestock species ranges between 2.5 to 3.5 microns and its length between 5 to 7 microns. It is known to contain several sperm-specific surface proteins which are not only unique, immunogenic, but also facilitate the binding of sperm to ovulated eggs [22,23]. The network of disulfide bonds in the sperm membrane is different and makes it more resistant to digestion when compared with other membranes [24]. This stability of the sperm membrane coupled with the presence of different sperm membrane specific proteins and other non-protein structures are an excellent candidate for aptamers selection. With respect to oligonucleotides, the robustness of the phosphodiester backbone enables them to exhibit improved stability over their protein-based antibody counterparts and also have shown greater potential in clinical trials and other *in-vivo* applications [25]. For use in aptamer generation, the length of the randomized region [number of nucleotides] in the oligonucleotide library is typically made in an arbitrary fashion [26] with the usual length between 40 to 70 nucleotides which allows greater complexity in the secondary and tertiary structures to be formed [27,28].

Hence, in this study, we designed an oligonucleotide library with a central 40 nucleotide (nt) random core flanked by 27 nucleotides to serve as primer binding sites (resulting in approx. 2.56 million types of oligonucleotides in the library) and used them in the Cell-SELEX to bind to bovine spermatozoa. A critical aspect in the SELEX rounds is the generation of single stranded DNA that forms the typical secondary structure and enables its binding specifically to the protein/structure of interest. Different approaches have been described to generate ssDNA, including asymmetric PCR [29], lambda exonuclease digestion [30] and alkali mediated separation [31,32]. In this study, a reasonable quantity of the required single strand of DNA was generated by asymmetric PCR and the SELEX rounds resulted in 257.8% increased oligonucleotide concentration at the end of the seventh round with an overall enrichment by 13.06-fold.

There could be a query on the generation of some amplicons across the SELEX rounds from non-specific primer binding to the bovine genomic DNA. Given the size of bovine genome to be 3 to 4 GB and a motif of approximately 10 nucleotides long is expected to occur at the rate of 43 times in the genome by chance. The genome of spermatozoa is highly condensed [33] and hence the genomic DNA may also be inaccessible for the oligonucleotides to bind in the conditions applied and hence lowering the possibility of enriching amplicons to sperm DNA [21]. In addition, the 27-nucleotide primer binding site on BLASTN analysis did not reveal any perfect or near perfect similarity to sequences in the bovine genome. This confirms that during the SELEX process, specific amplification of the spermatozoa bound oligonucleotides had taken place and there is no amplification from bovine DNA targets.

The effectiveness of the SELEX process is indicated by the reduction in the diversity of the oligonucleotide population over the SELEX rounds and the same can be confirmed by sequencing the SELEX round amplicons. When the whole cell approach was used to develop oligonucleotides in other studies which used 20 to 21 cycle in the PCR reaction, multiple oligonucleotides were also found after sequencing [34, 35] and only 10 per cent of 163 sequenced clones were found to be homologous [34]. It is therefore possible that sequencing fewer oligonucleotides clones from the SELEX rounds is not sufficient to identify homologous sequences in the final pool. In this context, the next generation sequencing (NGS) approaches would allow quick and massive parallelization of the sequencing process as well as offer greater sequencing coverage. The generated sequence data when coupled with specific bioinformatics tools would enable the clustering the vast range of sequences and identify potentially enriched oligonucleotides [36]. Generation of sequence data by NGS from different rounds of SELEX also provides the opportunity to assess the frequency of the oligonucleotides sequences and decide on the enrichment status even after fewer rounds of SELEX [36].

In this study, we generated paired end reads by NGS from the sixth and seventh rounds of SELEX to bovine spermatozoa and assessed their quality parameters using FastQC. We generated 14.11 and 17.38 million reads from sixth and seventh rounds of SELEX respectively with a Q30 value of 94 indicating extremely low chance of sequence base calling errors. Of the several tools available for clustering the NGS data, the CD-HIT [37] and FASTAptamer [38] have been used to determine the level of enrichment across SELEX rounds. The minimum number of identical short substrings, called ‘words’, shared by two sequences as a function of their sequence similarity was used to cluster the reads with a minimum of 90% similarity in CD-HIT. The FASTAptamer identifies a “seed” sequence for cluster generation indicating its abundance and calculates the Levenshtein edit distance based on the number of substitutions, insertions, or deletions necessary to transform a sequence into the seed sequence. Following this, the sequences are clustered together if the edit distance from the seed sequence is less than or equal to the edit distance specified (a distance of 4 was specified in this study). These two packages enabled effective clustering of the sequence reads and we narrowed down to 2,195 and 4,405 reads in the top three clusters in the sixth and seventh rounds, respectively.

The Mfold server enabled prediction of the secondary structures and the structural configuration in these oligonucleotides that might contribute to the spermatozoa binding [39,40]. The folding conditions for the oligonucleotide sequences were performed with the conditions represented in the binding buffer and all returned structural isoforms were analyzed for their ΔG values [41]. The ΔG values for the predicted structures ranged from 1.17 to −6.30 kcal/mol indicating a wide variety of stable structures. In addition, the MEME has been used in several studies to identify potential motifs [42,43] and in this study we identified 1 and 5 motifs from the sixth and seventh rounds of SELEX respectively. The first motif in seventh round of SELEX was present in 79% of the sequences (3,470 sequences out 4,405 sequences) and had an E value of 1.5e-42265. This is an interesting lead for the SELEX process followed as it confirms the contribution of the potential structures formed by such motif to bovine spermatozoa binding.

Hence, due to the above predicted features, we selected the top three oligonucleotides in the round 6, labelled with 5′ biotin and used for binding with bovine spermatozoa (~4 million spermatozoa) at three different concentrations (50, 100, and 200 pmol). The use of streptavidin coated magnetic beads enabled separation of the oligonucleotide bound spermatozoa and among the three oligonucleotides, the R6-Oligo-2 had a higher binding capability at 100 pmol concentration and resulted in trapping of almost 80% of the spermatozoa. The oligonucleotides from the seventh round of SELEX (cluster_1869, cluster_5, and cluster_2015) were labelled at their 5′ end with 6- FAM and this enabled us to determine the pattern of its physical binding to bovine spermatozoa.

By confirming the different binding patterns as well as the ability of the identified oligonucleotides to trap bovine spermatozoa, we successfully demonstrate an optimized process of cell-SELEX to spermatozoa and as a first step have generated aptamers with specific binding potential to bovine spermatozoa. The identification of oligonucleotide molecules with affinity to spermatozoa provides a new and quick approach to concentrate spermatozoa by selective trapping from complex samples (e.g. forensic samples) or from Oligospermic semen samples. Since this study has provided the basic strategy and also identified oligonucleotide pools with affinity to bovine spermatozoa, future competitive rounds of SELEX can be planned with X- or Y- enriched semen samples and possibly generate oligonucleotides with differential binding to bovine spermatozoa based on the chromosome type they carry. Generation of such molecules can help in reporting novel protocols/approaches with potential downstream applications to sort bovine spermatozoa

## Figures and Tables

**Table 1:** Concentration of the oligonucleotide library used and ssDNA yield in different rounds of SELEX

SELEX	Type of semen	Oligonucleotide library concentration used for SELEX^1)^	Oligonucleotide library concentration after SELEX^2)^	Increase in ssDNA oligo concentration in the SELEX Rounds (pmol/%)
SELEX I	Jersey Semen (Unsorted)	200 pmol oligo	1.39 ng/μL (total in 300 μL = 416 ng to 6.5 pmol)	-
SELEX – II	Jersey Semen (Unsorted)	6.5 pmol	2.02 ng/μL (total in 300 μL = 605 ng to 9.45 pmol)	2.95 (45.38)
SELEX – III	Jersey Semen (Unsorted)	9.45 pmol	8.00 ng/μL (total in 300 μL = 2,400 ng to 37.5 pmol)	28.05 (296.82)
SELEX – IV	Jersey Semen (Unsorted)	37.5 pmol	2.20 ng/μL (total in 300 μL = 660 ng to 10.31 pmol)	−27.91 (−72.06)
SELEX – V	Jersey Semen (Unsorted)	10.31 pmol	2.62 ng/μL (total in 300 μL = 787.5 ng to 12.30 pmol)	1.99 (19.30)
SELEX - VI	Jersey Semen (Unsorted)	12.30 pmol	5.45 ng/μL (total in 300 μL = 1,635 ng to 25.54 pmol)	13.24 (51.84)
SELEX-VII	Negative SELEX (diluents/tubes)	25.54 pmol	19.50 ng/μL (total in 300 μL = 5,850 ng to 91.4 pmol)	65.86 (257.87)

SsDNA, single-strand deoxyribonucleic acid; SELEX, systematic evolution of ligands by exponential enrichment.

1) In every round of SELEX, the spermatozoa (at a concentration of 2×10^8^) were bound with the different concentration of the oligonucleotide library in a total binding volume of 500 μL.

2) The concentration in ng was converted into pmol using online calculator available at http://www.promega.com/a/apps/biomath/

**Table 2:** Details on the sequences generated by Illumina HiSeq data from the sixth and seventh rounds of SELEX

Details	Round 6	Round 7
Number of raw reads	14,376,140	48,805,787
Number of quality passed reads	14,115,091	17,743,948
Number of CDHIT90 clusters	737,161	16,902,207
Number of reads in top 10 CDHIT 90 clusters	78,098	21,196
Number of unique reads in the top three clusters	15,701	3,113
Number of FASTAptamer collapsed reads	2,195	3,470
Number of unique reads in the top 10 clusters	16,673	4,405

SELEX, systematic evolution of ligands by exponential enrichment.
